# Asymmetric division: a marker for cancer stem cells?

**DOI:** 10.18632/oncotarget.1029

**Published:** 2013-05-10

**Authors:** Pengcheng Bu, Kai-Yuan Chen, Steven M. Lipkin, Xiling Shen

**Affiliations:** School of Electrical and Computer Engineering, and Department of Biomedical Engineering, Cornell University, Ithaca, NY, USA; School of Electrical and Computer Engineering, Cornell University, Ithaca, NY, USA; Departments of Medicine, Genetic Medicine and Surgery, Weill Cornell Medical College, New York, NY, USA; School of Electrical and Computer Engineering, and Department of Biomedical Engineering, Cornell University, Ithaca, NY, USA

Asymmetric cell division is a mechanism commonly used by stem cells in metazoan organisms to populate tissues and maintain homeostasis. During asymmetric division, a stem cell divides and generates a daughter stem cell for self-renewal and a daughter progenitor cell that undergoes further differentiation. In contrast to symmetric division, asymmetric division enables stem cells to self-renew and generate cellular diversity while mainitaing a constant number of stem cells, hence to prevent inadvertent depletion or overgrowth of the stem cell population. Consistent with this notion that asymmetric division regulates tissue cell census and homeostasis, disruption of asymmetric division in normal tissue often leads to dysplasia. Therefore, to maximize tumor cell proliferation, clonal evolution should favor cancer cells that perform symmetric division. Following this predominant line of thought, the question that whether cancer cells perform asymmetric division, and even if they do so, whether it holds any significance, has been largely overlooked.

The cancer stem cell (CSC) model kindled some interests in examining asymmetric division in cancer cells, because asymmetric division would provide a link between cancer and normal stem cells. Interestingly, recent reports have identified asymmetric cell division in breast, glioma, colorectal, and lung cancer, performed by a subpopulation of cells that share some stem-cell-like properties [1-5]. In these cells, the frequency of asymmetric division is negatively correlated with their proliferative capacity; namely, the more proliferative the cells became, the less asymmetric division and more symmetric division they performed. Studies in mouse models further demonstrated that decreased asymmetry in normal stem cells is associated with abnormal self-renewal and neoplastic transformation [6, 7]. Mechanistically, various factors including Akt, p53, EGFR and microenvironment signaling can affect the balance of cell fate choice between symmetry and asymmetry. Altogether, these studies raised 2 question: (a) why do some cancer stem cells still perform asymmetric division at certain frequencies even though asymmetric division is correlated with lower proliferative capacity, and (b) how are the different signaling cues integrated in cancer stem cells to determine cell fate asymmetry, which amounts to a binary decision?

To address these questions, Bu et al. [8] studied the balance between symmetric vs. asymmetric division in colon cancer stem cells (CCSCs) from early and late stage colorectal cancers (CRC). Pair-cell assays with immunofluorescence for CCSC and differentiation markers showed that asymmetric division happens frequently (12~19%) in early-stage CCSCs but rarely in late-stage CCSCs. Consequently, increased symmetric divisions in late stage CCSCs correlate with higher proliferative capacity and undifferentiated tumors. These observations suggest that asymmetric division is a marker for CCSCs from early-stage, likely more well-differentiated tumors, which may still preserve some of the original tissue hierarchy. It is tempting to postulate that this is due to the insufficient time for these early stage tumors to evolve away from their original tissue cell types. Not quite autonomous yet, these cells may still depend on some cellular hierarchy to survive in the tissue microenvironment. In contrast, late stage CRC cells have become more autonomous and function in an undifferentiated state, so asymmetric division is strongly selected against due to its lower proliferative capacity. This issue is important because stem cell properties generally are associated with late stage tumors. However, it is possible that certain stem cell line features (e.g. self-renewal to colonize metastatic sites) are associated with late stage poorly differentiated tumors, whereas others (e.g. asymmetric division and tumor organization similar to the tissue of origin) are more closely associated with early stage, better differentiated tumors.

Bu et al. further showed that the decision of symmetric vs. asymmetric division is regulated by a microRNA, miR-34a. The mir-34a level sits in a “sweet spot” to regulate asymmetric division in early stage CCSCs. Overexpression of miR-34a leads to symmetric self-renewal and proliferation whereas suppression of miR-34a leads to differentiation and depletion of CCSCs. Surprisingly, miR-34a appears to exert a stronger effect on the mode of division than the canonical cell fate determinant protein Numb, which had been used as a marker for asymmetry in previous studies. Investigation of the interaction between miR-34a and its target Notch1 revealed that miR-34a generates a bimodal Notch1 distribution, which produces binary cell fate decisions, whereas Numb regulates Notch1 in a more graded, continuous fashion. Besides targeting Notch (Notch1-2, JAG1, DLL1), miR-34a simultaneously target many genes that are involved in other aspects of cellular functions. Downstream of p53, miR-34a targets include BCL2 (apoptosis), CD44 (adhesion and migration), Myc, MycN (oncogene), SIRT1, HDAC1 (epigenetic modification), FOXP1 (pluripotency), WNT1, and MAP2K1 (proliferation). Therefore, the combination of its ability to generate binary response in its target genes and its ability to target many genes simultaneously makes miR-34a a master regulator of CCSC asymmetric division. miR-34a is suppressed in late stage CCSCs, leading to increased symmetric division and proliferation.

**Figure 1:**
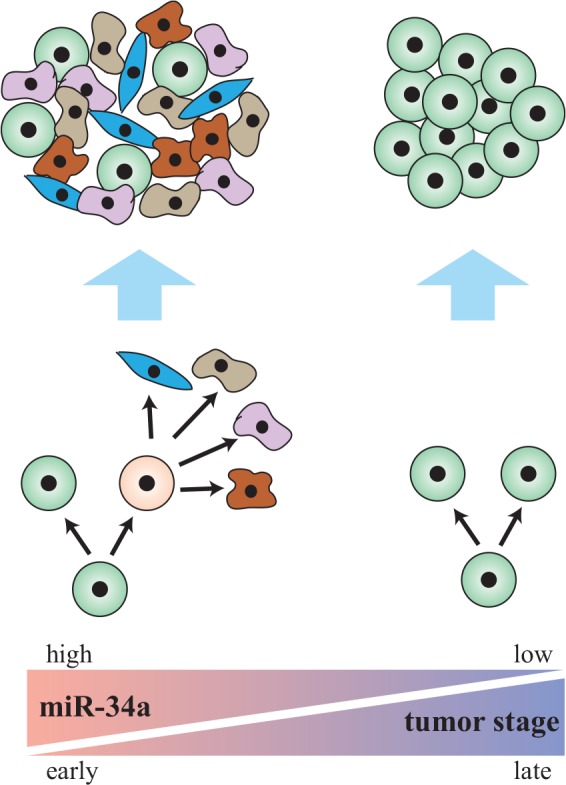
Asymmetric division is associated with cancer stem cells in early-stage tumors (Left) miR-34a regulates asymmetric division of cancer stem cells to form well-differentiated tumors in early-stage cancer. (Right) Late-stage cancer suppresses miR-34a and asymmetric division, which promotes symmetric self-renewal to form ∂undifferentiated tumors.

Altogether, the cited studies suggest that asymmetric division is a feature more closely associated with CSCs from early-stage, well-differentiated tumors. Late stage tumors suppress asymmetric division and increase symmetric division of self-renewing daughters to promote proliferation (Fig. 1). MicroRNAs may play a unique role in this decision, so restoration of its function in patients with late stage tumors may provide therapeutic benefits.
